# Development of a real-time endoscopic image diagnosis support system using deep learning technology in colonoscopy

**DOI:** 10.1038/s41598-019-50567-5

**Published:** 2019-10-08

**Authors:** Masayoshi Yamada, Yutaka Saito, Hitoshi Imaoka, Masahiro Saiko, Shigemi Yamada, Hiroko Kondo, Hiroyuki Takamaru, Taku Sakamoto, Jun Sese, Aya Kuchiba, Taro Shibata, Ryuji Hamamoto

**Affiliations:** 1 Endoscopy Division, National Cancer Center Hospital, Tokyo, Japan; 2 Division of Molecular Modification and Cancer Biology, National Cancer Center Research Institute, Tokyo, Japan; 3 Biometrics Research Laboratories, NEC Corporation, Kanagawa, Japan; 4 Advanced Intelligence Project Center, RIKEN, Tokyo, Japan; 5 Artificial Intelligence Research Center, National Institute of Advanced Industrial Science and Technology, Tokyo, Japan; 6 Biostatistics Division, National Cancer Center, Tokyo, Japan

**Keywords:** Cancer screening, Colonoscopy, Mechanical engineering

## Abstract

Gaps in colonoscopy skills among endoscopists, primarily due to experience, have been identified, and solutions are critically needed. Hence, the development of a real-time robust detection system for colorectal neoplasms is considered to significantly reduce the risk of missed lesions during colonoscopy. Here, we develop an artificial intelligence (AI) system that automatically detects early signs of colorectal cancer during colonoscopy; the AI system shows the sensitivity and specificity are 97.3% (95% confidence interval [CI] = 95.9%–98.4%) and 99.0% (95% CI = 98.6%–99.2%), respectively, and the area under the curve is 0.975 (95% CI = 0.964–0.986) in the validation set. Moreover, the sensitivities are 98.0% (95% CI = 96.6%–98.8%) in the polypoid subgroup and 93.7% (95% CI = 87.6%–96.9%) in the non-polypoid subgroup; To accelerate the detection, tensor metrics in the trained model was decomposed, and the system can predict cancerous regions 21.9 ms/image on average. These findings suggest that the system is sufficient to support endoscopists in the high detection against non-polypoid lesions, which are frequently missed by optical colonoscopy. This AI system can alert endoscopists in real-time to avoid missing abnormalities such as non-polypoid polyps during colonoscopy, improving the early detection of this disease.

## Introduction

The incidence of colorectal cancer (CRC) has been increasing both in Japan and globally^1,2^. In Japan, more than 130,000 people were diagnosed with CRC in 2013, and more than 50,000 people died of the disease in 2016^1^. Importantly, colonoscopy following the removal of detected neoplastic lesions reduces the incidence and mortality of CRC^3,4^. Therefore, it is essential to perform colonoscopy; however, the incompleteness of colonoscopy may lead to post-colonoscopy CRC (PCCRC), a recent problem of colonoscopy. PCCRC has been reported to account for 3%–10% of all resected CRC lesions^5,6^. The reasons for PCCRC include missed lesions (58%), failure to visit the hospital (20%), newly occurring lesions (13%), and residual lesions due to inadequate endoscopic treatment (9%)^7,8^. Several studies described the characteristics of PCCRC as follows: (1) right-sided colon location, (2) small and early-stage cancer, and (3) flat morphology^8–10^. Indeed, the missed polyp rate during colonoscopy has been reported as approximately 20%, but the rate varies according to the skill of the endoscopist^11,12^. Hence, we hypothesized that artificial intelligence (AI) technology may help prevention of missed lesions during colonoscopy and reduce the skills gap among endoscopists, particularly regarding the detection of flat lesions.

AI technology, which is defined as the science and engineering of creating intelligent machines, has greatly progressed in recent years, primarily due to the advancement of analysis methodologies such as neocognitron, support vector machine, and deep learning^13^. Deep learning, also known as deep structured learning or hierarchical learning, is part of a broader family of machine learning methods based on learning data representations. In 2006, Hinton and colleagues described the use of the generalized backpropagation algorithm to train multilayer networks, which led to the breakthrough of deep learning^14^. Deep learning architectures are known to be particularly suitable for quantifying images, exhibiting high capability in detection, classification, and segmentation^15^. In fact, AI using deep learning achieved and exceeded human-level image recognition in a competition at the ImageNet Large Scale Visual Recognition Challenge in 2015 (error rate: 4.9% vs. 5.1%)^16^. AI systems using deep learning have been applied for images of lesions such as breast cancer, skin cancer, and diabetic retinopathy, and the systems are appropriate for image feature analysis^17–19^. Moreover, AI systems have been used in mammography and computed tomography, albeit on an extremely limited basis^20^. Although AI has been applied to polyp detection in colonoscopy, the results have not been satisfying^21^. In the present study, we developed an AI system that automatically detects early signs of CRC during colonoscopy on an almost real-time basis.

## Results

**Figure 1:**
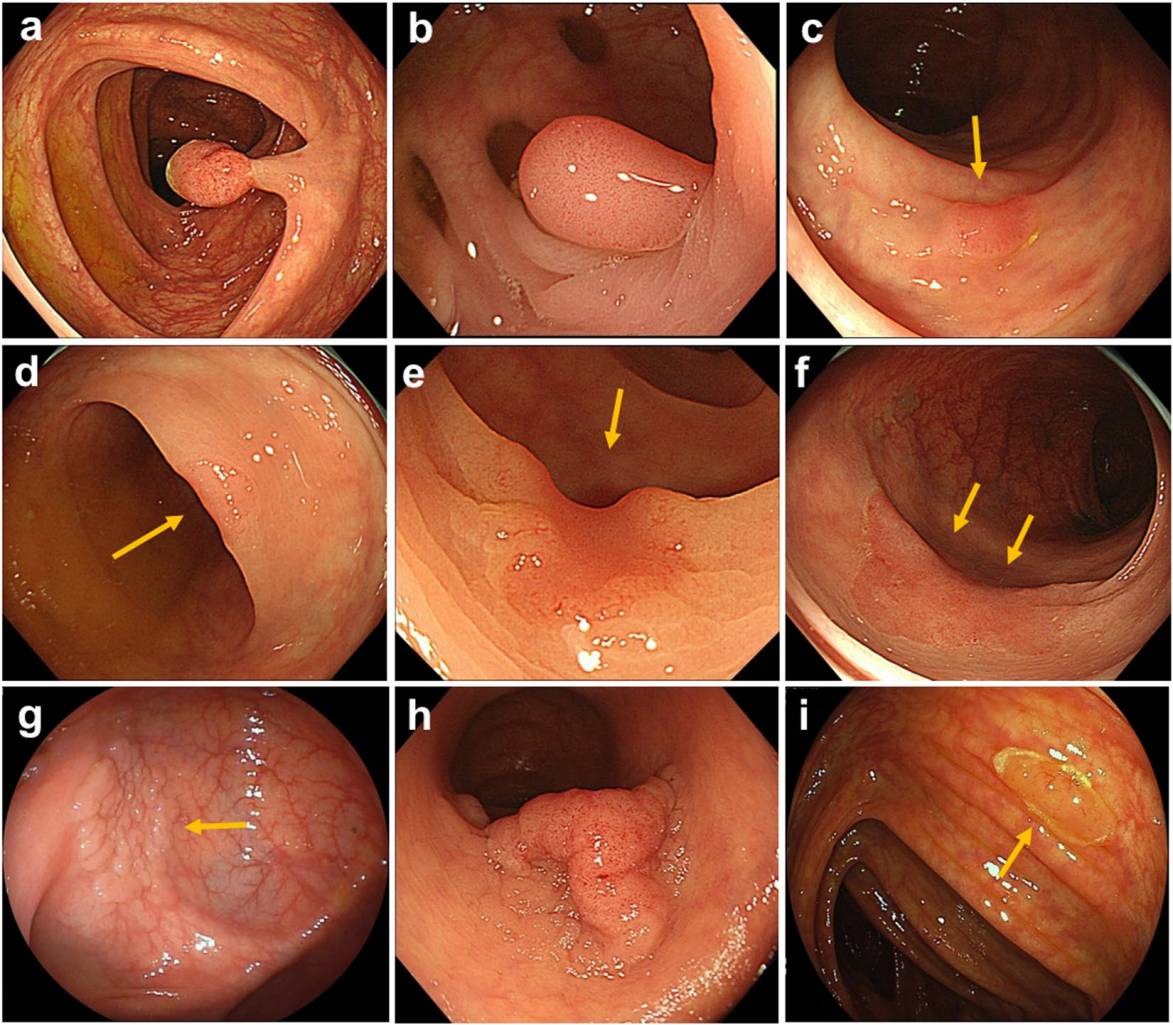
Representative images of trained colonic lesions. (**a**) 10-mm sized pedunculated type. (**b**) 10-mm sized sessile type. (**c**) 4-mm sized superficial elevated type. (**d**) 4-mm sized superficial depressed type. (**e**) 4-mm sized superficial depressed type. (**f**) 25-mm sized non-granular type laterally spreading tumor. (**g**) 18-mm sized granular type laterally spreading tumor. (**h**) 50-mm sized granular type laterally spreading tumor. **i**, 6-mm sized sessile serrated lesion.

We started training using three groups of images: group 1, 1,244 still images of 1,379 polypoid lesions; group 2, 891 frames of 173 consecutive lesions and 134,983 frames of noncancerous tissue from videos; and group 3, 2,843 still images of 564 slightly elevated and depressed lesions (Fig. 1). Each image was transformed to be a resolution of 880 × 752 pixels after cropping area of endoscopy image from display screen image, since the area size of endoscopy image is frequently changed. The size, 880 × 752 pixels, was experimentally determined based on our experimental images.

All lesions in the training and validation sets were pathologically proven early-stage CRCs (Tis or T1) or precursor lesions (Table 1). Histological diagnosis was performed using the World Health Organization criteria. In the present study, precursor lesions included adenoma, dysplasia, and sessile serrated adenoma/polyps (SSA/Ps) (Fig. 1). We included images of hyperplastic polyps (HPs) in the right-sided colon (from the cecum to descending colon) in the training set because interobserver agreement among pathologists for discriminating HPs and SSA/Ps was reported to be challenging in histology^22^. Furthermore, including images of HPs in the right-sided colon is consistent with the clinical recommendation in the National Comprehensive Cancer Network guideline^23^. In group 2, we included 33 images of eight consecutive patients with advanced CRC (Borrmann classification type 2) to prevent missing intermediate lesions. All lesions in the training set images were manually annotated as regions of interest (ROIs) at their edges by experienced endoscopists (Supplementary Fig. 1). The extracted ROIs were categorized as positive samples and regions outside the ROIs were deemed negative samples in the supervised deep learning model. The regions of negative samples were selected randomly.

**Table 1:** Clinicopathological characteristics of lesions in the validation set.

	Still image	Video image
Number of images or videos validated	4,840 images	77 videos
Number of endoscopists, n	15	14
Number of lesions (images or videos)	752 (702)	56 (45)
**Location of lesions**		
Right-sided colon	351 (47%)	33 (59%)
Left-sided colon	254 (34%)	20 (36%)
Rectum	147 (19%)	3 (5%)
Size of lesions, mm, median, IQR	5 (4–10)	4 (3–5)
**Morphological type, n (%)**		
Polypoid	638 (85%)	12 (21%)
Slightly elevated and depressed	114 (15%)	44 (79%)
**Pathological diagnosis, n (%)**		
Hyperplastic polyp	23 (3%)	3 (5%)
Sessile serrated adenoma/polyp	40 (5%)	3 (5%)
Traditional serrated adenoma	9 (1%)	0
Low-grade adenoma/dysplasia	441 (59%)	47 (84%)
High-grade adenoma/dysplasia	214 (28%)	2 (4%)
Submucosal invasive cancer	25 (3%)	1 (2%)

IQR, interquartile range.

Right-sided colon includes cecum, ascending colon and transverse colon; Left-sided colon includes descending colon and sigmoid colon; Rectum includes rectsgmoid colon, upper rectum and lower rectum.

Polypoid type includes 0-Is, Isp, Ip, granular type laterally spreading tumor (LST-G) nodular mixed type; Slightly elevated and depressed includes 0-IIa, IIc, LST-G homogenous type and non-granular type LST (LST-NG).

The deep learning model, which consists of supervised neural networks with multiple layers, has been successfully applied to a variety of computer vision tasks^24,25^. To detect lesions from endoscopic video frame images in the present study, our lesion detection model was Faster R-CNN with VGG16, which is one of the frequently used deep neural network models for object recognition^26,27^. This model combines two models: a classifier model for lesion detection and a regression model for lesion position (Supplementary Fig. 2). The classifier model is a binary classifier for lesions that outputs confidence scores for lesions. The regression model is a linear regression model that outputs the predicted positions of lesions. Each model shares the same feature extractor^28^. Using 9 types of multiscale sliding windows in accordance with the original study^27^, these two models detect lesions of various sizes. Both models were trained using stochastic gradient descent algorithms. The learning rate gradually decreased from 0.001 to 0.0001. However, the prediction speed is not so enough fast for endoscopists to use the model in real-time examination. Hence, by adopting the tensor decomposition method of Kim *et al*. to the trained model, the number of weight parameters was 5 times fewer, and the prediction speed was increased 1.7 times faster than original model by keeping the original accuracy^29^.

The diagnostic performance of the AI system was confirmed using the validation set (705 still images of 752 lesions and 4,135 still images of noncancerous tissue). The clinicopathological characteristics of the lesions in the validation set are shown in Table 1. The polypoid subgroup included 48 granular-type laterally spreading tumors, nodular mixed type. The superficial type included nine granular-type laterally spreading tumors, homogenous type and 30 nongranular-type laterally spreading tumors. The AI system and its user were blinded to the absence or presence of lesions and clinical information.

The AI system output was independently checked by three reviewers (MY, SY, and HK). The output for the lesion was considered correct when the system detected and flagged the lesion locus in. The output for no lesion area in the image with the lesion was considered correct when the all three observers didn’t detect any lesions outside the flag. The output for image without lesion was considered correct when the AI system showed no flag. The review process was conducted by three reviewers with knowledge of the clinicopathological data of the image. When a disagreement occurred among three reviewers, it was settled by discussion and all reviewers finally reach the common conclusion for all cases^30^. The sensitivity and specificity of the AI system were calculated.

**Figure 2:**
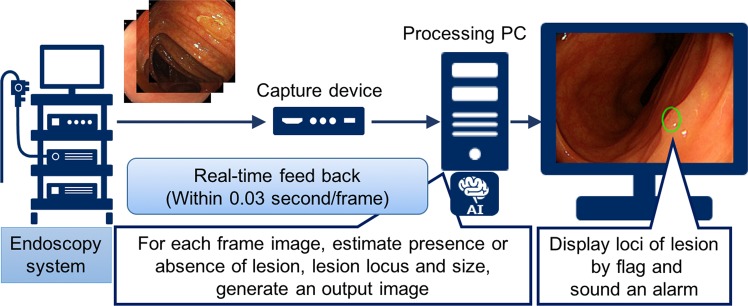
Represented schematically outline of the developed artificial intelligence system.

**Figure 3:**
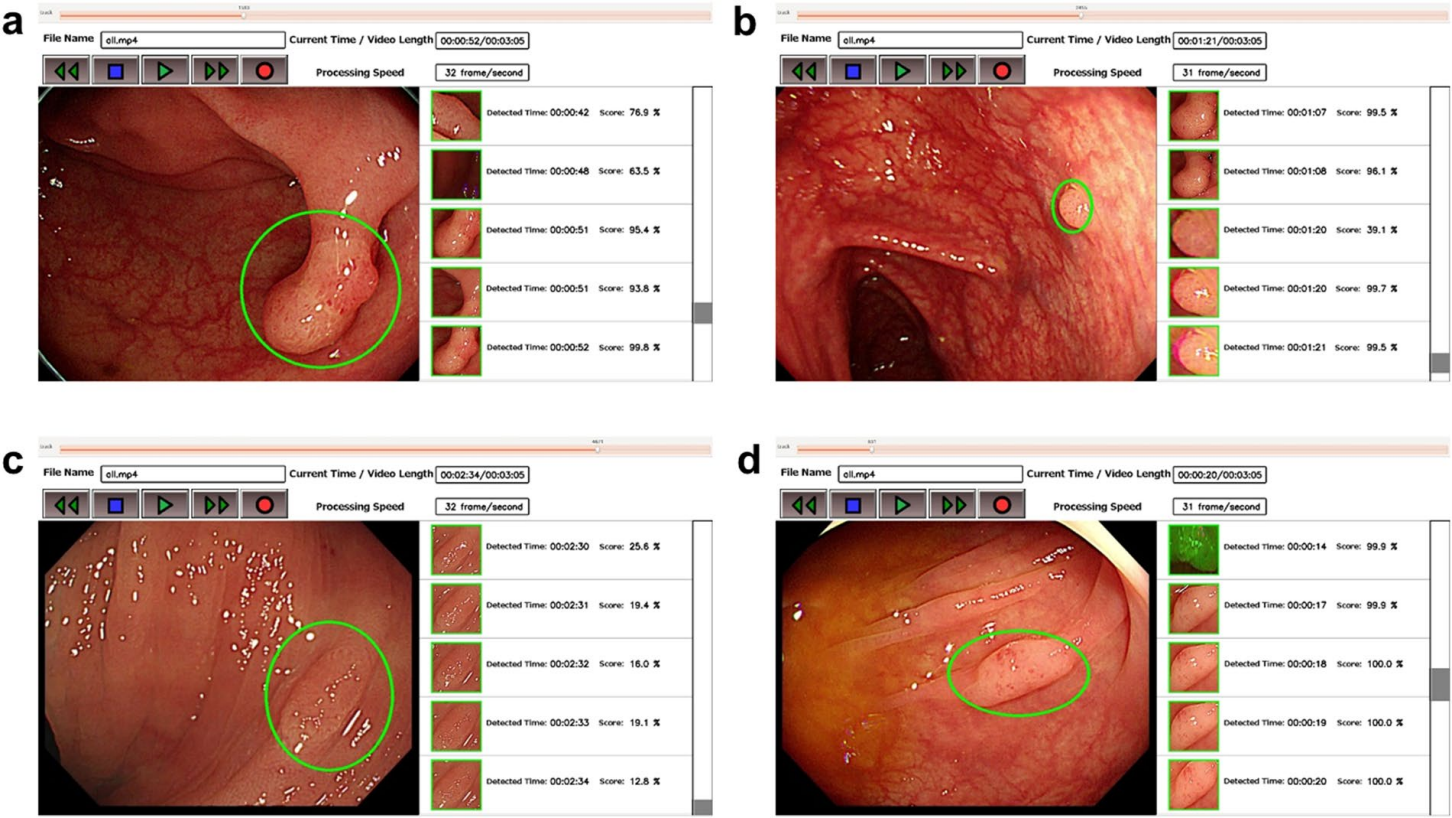
Representative images of detected polyps. (**a**) A 10-mm adenomatous polyp (polypoid type). (**b**) A 2-mm adenomatous polyp (polypoid type). (**c**) A 4-mm adenomatous polyp (slightly elevated type). (**d**) A 5-mm serrated lesion (slightly elevated type).

**Figure 4:**
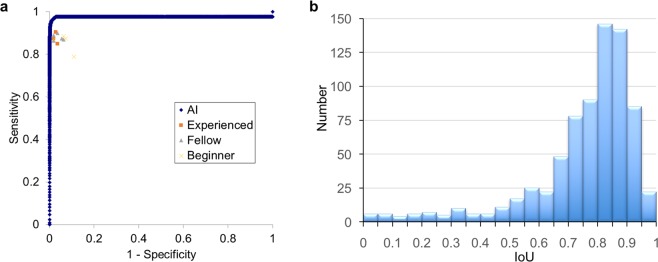
Comparing diagnostic performance between the AI system and endoscopists, and Intersection over the union (IoU) for the lesion detection. (**a**) Diagnostic performance was represented by the receiver-operating characteristic curve with AUC = 0.9752. Each orange, gray, and yellow point represents the sensitivity and specificity of an endoscopist. (**b**) If we defined poor = IoU < 0.5, good ≥0.5, <0.7, excellent ≥0.7, Good and Excellent was 91%, indicating AI flag is almost correct for lesions detection.

The AI system exhaustively analyzed each frame or image, detecting and displaying a result within 0.03 s (30 frames/s) (Fig. 2; Supplementary Video 1); representative images of detected polyps are shown in Fig. 3. In the validation study, the sensitivity and specificity of the AI system were 97.3 (95% CI = 95.9–98.4) and 99.0% (95% CI = 98.7–99.3), respectively (Table 2). In subgroup analysis, the sensitivity was 98.1% (95% CI = 96.8–99.0) in the polypoid subgroup, versus 92.9% (95% CI = 86.4–96.9) in the nonpolypoid subgroup. The area under the ROC curve was 0.9752 (95% CI = 0.964–0.986), and a supplementary human observational study demonstrated that the AI system had a superior diagnostic yield as endoscopists, including experienced, fellows, and beginners (Fig. 4a and Table 3). In all endoscopists, the sensitivity and specificity were median 87.4% (range 78.9–90.5) and 96.4% (range 89.1–98.2), respectively. The sensitivity was almost equal between experts, fellows, and beginners [87.4% (84.9–90.5), 87.4% (86.4–89.9) and 87.1% (78.9–88.4), respectively], whereas the specificity was high depending on the experience [experts, fellows, and beginners; 97.3% (96.4–98.2), 96.4% (93.6–98.2), and 93.2% (89.1–98.2), respectively]. The AI system analyzed all 4,840 images in 106.0 s (average, 21.9 ms/image), whereas endoscopists required median 725.2 s (IQR = 655–914) to analyzed the 309 images (median, 2.4 sec/image).

**Table 2:** Diagnostic performance of AI system for detecting and displaying early stage colorectal cancers and precursor lesions in still images.

	Sensitivity*, (n) (95% CIs)	Specificity^†^, (n) (95% CIs)	
		With lesions	Without lesions
All lesions (752 lesions)	97.3% (732/752) (95.9–98.4)	90.9% (638/702) (88.5–92.9)	99.0% (4094/4135) (98.7–99.3)
Polypoid lesions (640 lesions^‡^)	98.1% (628/640) (96.8–99.0)	90.4% (535/592) (87.7–92.6)	—
Superficial lesions (112 lesions^‡^)	92.9% (104/112) (86.4–96.9)	95.9% (93/97) (89.8–98.9)	—

*Sensitivity was defined as AI correctly detected lesion number/number of all lesions; ^†^Specificity was defined as AI negative image number/true lesion negative image number (images without lesions); Correct answer was defined when AI detect and display loci of lesion by flag when the all three observers didn’t detect any lesions outside the flag or no flag, or when AI detect and display no loci when the image shows truly no lesion. ^‡^Since 13 images included both polypoid and superficial lesion, they were excluded from the subgroup specificity analysis (with lesions).

**Table 3:** Diagnostic performance of the artificial intelligence (AI) system and endoscopists for detecting early-stage colorectal cancer and precursor lesions.

	All endoscopists n = 12	Experts n = 3	Fellows n = 5	Beginners n = 4	AI
Sensitivity	87.40%	87.40%	87.40%	87.10%	97.30%
median (range)	(78.9–90.5)	(84.9–90.5)	(86.4–89.9)	(78.9–88.4)	(95.9–98.3)
Specificity	96.40%	97.30%	96.40%	93.20%	99.00%
median (range)	(89.1–98.2)	(96.4–98.2)	(93.6–98.2)	(89.1–98.2)	(98.6–99.2)
Processing time	2.4 sec/image	2.7 sec/image	2.2 sec/image	2.4 sec/image	0.022 sec/image
median (range)	(1.5–12.9)	(2.1–4.7)	(1.5–8.7)	(1.7–2.9)	

AI, artificial intelligence. Endoscopists were tested 309 images while AI did 4840 images.

Accuracy of the AI flag localization was shown using intersection over the union (IOU) (Fig. 4b). If we defined poor = IoU < 0.5, good ≥0.5, <0.7, excellent ≥0.7, Good and Excellent was 91%, indicating AI flag is almost correct for lesions detection. Representative images of various IOUs were shown in the Supplementary Fig. 3.

Moreover, a comparison between the rectangle size of the flag and confidence score in the validation set illustrated that the confidence score varied greatly in the small rectangle size of the flag (Supplementary Fig. 4). Representative images of various rectangle sizes of the flags were shown in the Supplementary Fig. 4. Data from the images with lesion suggests that the AI system tend to detect the lesions when the rectangle size of the flag is large.

The high diagnostic performance was also validated in the independent video image sets (Table 4). If we defined the sensitivity and specificity as correct frame number in all frame number (definition 1), the sensitivity, specificity, false negative rate (FNR), false positive rate (FPR) with lesion and FPR without lesion was median 74.0% (interquartile range 47–85), 94.5% (89–98), 26.1% (15–53), 1.2% (0.1–8.7) and 5.5% (1.9–10.6), respectively. If we define the sensitivity as lesions that the AI system correctly detect the lesion consecutive 5 or more frame one time or more, and specificity as consecutive 5 or more correct frame number in all frame number (definition 2), these values are 100% (57/57 lesions), 98.0% (94.6–99.5), 0% (0/57 lesions), 0% (0.0–28.7) and 2.0% (0.5–5.4), respectively. If we define the sensitivity and specificity as correct when 50% or more of the entire frame is a correct frame, and calculate number of correct videos or correct number of lesions (definition 3), these values are 70.2% (40/57 lesions), 100% (77/77 colonoscopies), 29.8% (17/57 lesions), 0% (0/57 lesions) and 0% (0/77 colonoscopies), respectively. Although the value varies depends on the definition in the video analysis, the strictest definition 1 even had the high diagnostic performance. Furthermore, scatter diagrams of sensitivity and specificity divided for each endoscopy vendor illustrated that distribution of the plots was resembling regardless of the endoscopy vendor (Supplementary Fig. 5).

**Table 4:** Diagnostic performance of AI system for detecting and displaying early stage colorectal cancers and precursor lesions in 77 videos frames.

	With lesions^§^		Without lesion
**AI flag**	**Sensitivity**	**False positive**	**False positive**
Present	74.6% (47.0–85.1)	1.1% (0.1–8.3)	5.5% (1.9–10.6)
Def. 1*	100% (56/56 lesions)	0% (0.0–1.6)	2.0% (0.5–5.4)
Def. 2^Ɨ^	69.6% (39/56 lesions)	1.8% (1/56 lesions)	0% (0/77 colonoscopies)
**Def**. **3**^**ǂ**^			
**Absent**	**False negative**	—	**Specificity**
Def. 1*	25.4% (14.9–53.0)		94.5% (89.4–98.1)
Def. 2^Ɨ^	0% (0/56 lesions)		98.0% (94.6–99.5)
Def. 3^ǂ^	30.4% (17/56 lesions)		100% (77/77 colonoscopies)

Data shows median (interquartile range).

*Definition 1 = correct frame number/all frame number.

^Ɨ^Definition 2 = consecutive 5 or more correct frame number/all frame number.

^ǂ^Definition 3 = correct when 50% or more of the entire frame is a correct frame, and calculate number of correct videos/number of lesions.

^§^56 colonic lesions were included in the 77 videos.

Def., definition.

## Discussion

In this study, we developed a real-time endoscopic image diagnosis support system using deep learning technology in colonoscopy. Although prior studies attempted to develop AI systems for detecting gastrointestinal tumors^31–38^, the diagnostic performance and processing speed were unsatisfactory to use in real-time. Recent two studies using deep learning technology for detection of colorectal polyps reported meaningful and valuable data, however, detection for non-polypoid lesions is unclear^39,40^. This is a clinically critical question because the non-polypoid lesion is a target lesion in this kind of AI system that support human physician because we can detect polypoid lesions easily. The strengths of our AI system include (1) its high diagnostic performance using approximately 5,000 images of more than 2,000 lesions, (2) the inclusion of approximately 3,000 images of more than 500 non-polypoid superficial lesions in the training set, and (3) its nearly real-time processing speed. These results demonstrate that this AI system can be used to provide real-time feedback to physicians in clinical practice.

Given that we aim to use the AI system during colonoscopy without interrupting any doctors’ operations, we developed the real-time system, which enables fast detection. In addition, the principal aim of this AI system was to prevent missed lesions during colonoscopy; therefore, superior sensitivity and specificity are required, compared with those archived standard endoscopists. The sensitivity of our AI system for diagnosing early-stage CRC and precursor lesions was consistent with recently reported data for deep learning in skin cancer and diabetic retinopathy^18,19^. With regard to the specificity, high specificity is needed because it is extremely difficult to perform colonoscopy twice for the same patient due to the bowel preparation procedures. The validation study was conducted using images from three major endoscopy vendors, and there was no significant difference in the AI diagnostic performances among the three vendors. Therefore, we considered that the developed AI model could be used vendor-free when we train the AI model more. Further, because video images have a resolution of 30 frames/s, if the specificity is low, the high false-positive rate will be an obstacle to its use in colonoscopy. The present data illustrates that the AI system we developed is an ideal tool to use in colonoscopy.

Another expected benefit of the developed system is to improve the quality of colonoscopy. Corley *et al*. reported that a 1.0% increase in the adenoma detection rate expected a 3.0% decrease in the risk of PCCRC^6^. However, the quality of colonoscopy is usually affected by the skills gap among endoscopists. Rex *et al*. previously reported a polyp miss rate of 17%–48% in a tandem study^11^. This AI support system is expected to improve the detection of neoplastic colorectal polyps and equalize the quality of colonoscopy. Additionally, the AI system can comprehensively analyze whole endoscopic images, which compensates for the limitations of the human field of vision, and reduces the risk of missed polyps. This type of AI system is likely to be applicable for wide field-of-view endoscopy, a recent technological advancement in colonoscopy^41–44^. Indeed, it was reported that the devices provide up to 330° of view could improve the adenoma miss rate as large as 34%^44^. Moreover, the number of monitors used by endoscopists has also been increasing. Even under multiple monitors, this AI system is possible to sufficiently support endoscopists because of compensating for the limitation of the human field of vision.

The lack of robust computations have limited the utility of computer-aided diagnosis systems for decades^45^. As a consequence, we planned to set several stages to obtain a robust computation as follows: (1) learning still and video images from consecutive patients, (2) learning images captured from a high number of endoscopists (more than 30 endoscopists), and (3) learning slightly elevated and depressed lesions, which have a low prevalence among colorectal tumors^46^. A robust AI system can potentially overcome gaps in colonoscopy skills among physicians, and the expanded use of our developed system; for instance, the application by utilizing computer clouds may enable the global use of the AI support system at low cost.

We used Faster R-CNN model that is one of the two-stage detectors for lesion detection model even though we know that some one-stage detector like YOLO could be also available^47^. The reason why we did not use the YOLO algorithm is that one-stage frameworks typically show much poorer performance on detecting small objects than two-stage architectures^48,49^. Such characteristic is undesirable for lesion detection. In addition, given that the lesions do not intersect during the colonoscopy examination, we conclude that tracking procedures are not necessary from the viewpoint of clinical applications. For the above reasons, we consider that it’s critically important to detect early-stage small lesion. To train the Faster R-CNN model for lesion detection, we used a Faster R-CNN model trained with ImageNet dataset as a pre-trained model, and then the pre-trained model was fine-tuned with group 1 and 2 images (1,244 still images of 1,379 polypoid lesions, 891 frames of 173 consecutive lesions and 134,983 frames of noncancerous tissue from videos). The pre-trained model, trained for 1,000 object category recognition task of ILSVRC2012, can extract a kind of universal features such as edges and curves^50^. Among those features, some effective features for lesion detection are enhanced during the fine-tuning procedure. This transfer learning technique makes it possible to train high accuracy lesion detection model, while it’s generally hard to train models from scratch using only 2,135 images of lesions and 134,983 frame images of noncancerous tissue.

As for the issues of the system we developed in this study. this AI system failed to recognize 20 lesions in the images (false-negative rate of 2.7%). These missed lesions were captured obliquely along the edges of the images, or they were hidden by the haustra of the colon, indicating that most of them will be detected when this AI system is used in real time *in vivo* with careful observation. Furthermore, on the basis of the relationship between the rectangle size and confidence score, this AI system has weak performance to detect lesions in the distant areas of the image. This is reasonable because the lesion images used in the training set were captured when the endoscopists were aware of the presence of a lesion in the clinical setting. Therefore, we used consecutive video images in the second training period. Further accumulation of lesion images for training, including those in distant areas, may establish this AI system as a clinically available real-time AI support system.

In fact, this study was limited by its retrospective design. Additionally, we used consecutive lesions in one training period and in the validation set; the images in the training and validation sets were captured in a single high-volume center. However, more than 30 endoscopists captured the images, and one advantage of this AI system is its applicability to a number of endoscopes developed by two major distributors (Olympus Optical and Fujifilm Medical). Thus, this AI system must be more robust than other reported systems. Moreover, although there was no *in vivo* validation data in this study, we confirmed that the resolution of images was 30 frames/s, and that this AI system had high accuracy using video images. In addition, we are going to start *in vivo* clinical trials using this AI system.

In conclusion, we have developed an AI system that automatically detects early signs of CRC during colonoscopy. This AI system can alert doctors to abnormalities such as polyps in real time during colonoscopy, allowing for improved early detection of the disease.

## Methods

### Patients and colonoscopy image samples

This study was approved by the Ethics Committee of the National Cancer Center, Tokyo, Japan. All methods were performed in accordance with the Ethical Guidelines for Medical and Health Research Involving Human Subjects. Informed consent was obtained from each patient included in the study. All colonoscopic still and video images were obtained at this institution. We retrospectively collected images of colonoscopies performed between January 2015 and June 2016. The images were assigned to the training set of the deep learning model (obtained between January 2015 and April 2016) or the validation set (obtained between May 2016 and June 2016). All images were obtained using standard endoscopes (PCF-Q240ZI, CF-H260AZI, PCF-Q260AZI, CF-HQ290AI, or PCF-H290AZI; Olympus Optical Co., Tokyo, Japan and EC-580RD/M, EC-590MP, EC-590ZP, EC-590WM3, EC-600ZW/M, EC-600WM, EC-L600ZP; Fujifilm Medical Co., Tokyo, Japan) and a standard video processor system (EVIS LUCERA system; Olympus Optical; Advancia HD or LASEREO system; Fujifilm Medical).

### Real-time endoscopic image diagnosis support system

To adapt the devised program to colonoscopy, we developed an AI system. Specifically, the video image signal from the video processor system was input into a personal computer with graphics processing units (NVIDIA Geforce GTX 1080 × 2) via a capture device that converts the signal into image data. The computer program runs all video frames (30 frames/s) and exhaustively analyzes each frame. When the AI system detects a lesion, it flags the locus and provides an audio alert.

### Validation of the AI system

To compare diagnostic yields between the AI system and endoscopists, an observation study was performed using randomly selected images from the validation set (199 images with lesions and 110 images without lesions) which is independent of the training set. A written informed consent was obtained from all participated endoscopists. The endoscopists were classified into experienced (≥5,000 colonoscopies, three endoscopists), fellow (<5,000 colonoscopies and certification by the Japan Gastroenterological Endoscopy Society, five endoscopists), and beginner groups (<5,000 colonoscopies and no board certification, four endoscopists). The observers were blinded to both the histopathological diagnosis and clinical information, and the images were evaluated randomly to calculate the human diagnostic yield for each observer.

### Statistical analysis

The performance of the developed AI system was evaluated by estimating the sensitivity and specificity with their Clopper-Pearson exact 95% confidence intervals (CIs). The flag was set to display the locus when the confidence score exceeded 0.1. The sensitivity was defined on a lesion-basis and estimated as the proportion of AI correctly flagged lesions among the pre-defined lesions. The specificity was defined on an image basis. We estimated two types of specificity: one was the proportion of no flag images among the images without lesions, the other was the proportion of the images with no flag within no lesion region among the images with lesions.

We also calculated a receiver-operating characteristic (ROC) curve and the area under the curve (AUC) based on the different cutoffs of confidence scores of the AI system for each image with or without lesions using the validation set. The first specificity above was used for ROC analysis.

To validate accuracy of the AI flag localization, we calculated intersection over union (IoU). IoU demonstrates the rate of correct area in entire area of the flag (ground truth and AI flag). If there were two or more AI flag in one image, AI flag with highest confidence score was chosen for this IoU analysis.

## Supplementary information

Supplementary information

Supplementary Video 1
