# Supplementary material for: Development of a real-time endoscopic image diagnosis support system using deep learning technology in colonoscopy
Source: Sci Rep. 2019 Oct 8;9:14465. doi: 10.1038/s41598-019-50567-5 (PMC6783454; doi:10.1038/s41598-019-50567-5)
Supplement: Supplementary file 1 — Supplementary information [file 41598_2019_50567_MOESM1_ESM.docx]

**Supplementary information**

**Development** **of a real-time endoscopic image diagnosis support system using deep learning technology in colonoscopy**

Masayoshi Yamada, Yutaka Saito, Hitoshi Imaoka, Masahiro Saiko, Shigemi Yamada^2,4^, Hiroko Kondo, Hiroyuki Takamaru, Taku Sakamoto, Jun Sese, Aya Kuchiba, Taro Shibata, Ryuji Hamamoto

The file contains

Supplementary Figures 1 – 5

**Supplementary Figure 1 │ The training set images were manually annoted as ROIs.** The extracted ROIs were categorized as positive samples and refions outside the ROIs were deemed negative samples in the supervised seep learning model.

**Supplementary Figure 2 │ Faster R-CNN with VGG 16 model is used.** This model has both a classifier model for lesion detection and a regression model for lesion position.

**Supplementary Figure 3 │ Representative images of relationship of AI detected and correct flag.** The blue flag is AI detected flag and green is correct flag supervised by endoscopist. The intersection over union (IoU) is 0.01 (**a**), 0.70 (**b**), and 0.87 (**c**).


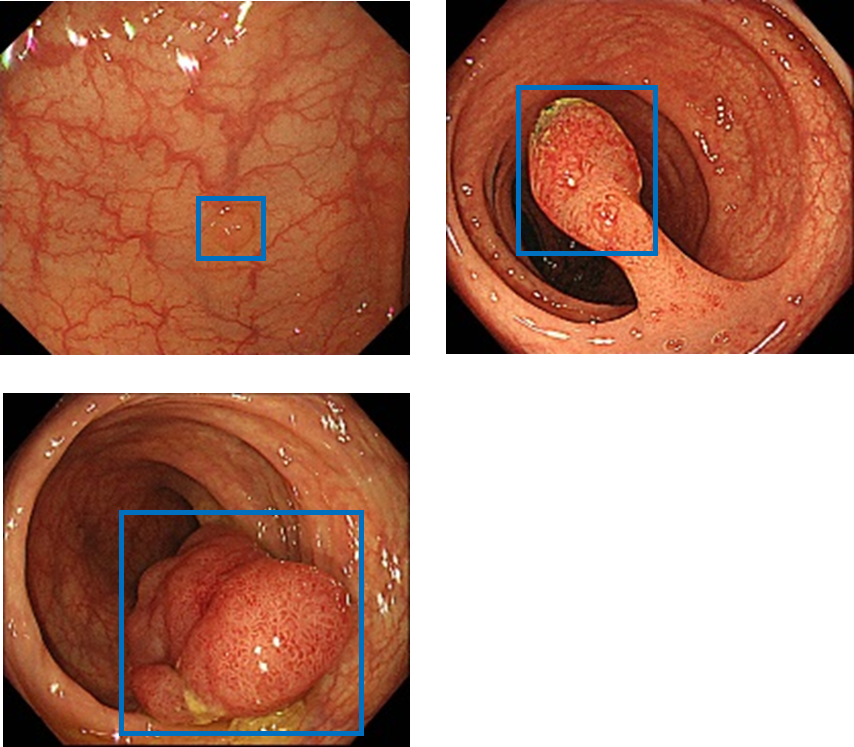


a

b

c

d

**Supplementary Figure 4 │ Comparison between the rectangle size of the flag and confidence score.** When the flag size is large, confidence score is high. But when the flag size is small, confidence score is also low (a), Representative images of flag sizes (pixels wide* height). The flag size is 12,320 pixcels (**b**), 109,921 pixels (**c**) and 226,440 pixels (**d**).

**Supplementary Figure 5 │ Difference of diagnostic yield between endoscopy vendors**. Distribution of the plots of both sensitivity (**a**) and specificity (**b**) seems resembling.
